# Bifid cardiac apex and spongiform cardiomyopathy in fetus with small microdeletion 16p12.2 of paternal origin. Critical points in family communication on 16p12.2 microdeletion

**DOI:** 10.1002/ccr3.7602

**Published:** 2023-07-02

**Authors:** Mariano Stabile, Anna F. Rispoli, Maurizio Capuozzo, Umberto Ferbo, Guglielmo Stabile

**Affiliations:** ^1^ Zygote Center: Center for Genetics—Prenatal Diagnosis—Fertility Salerno Italy; ^2^ Centro Direzionale di Napoli Innovalab Scarl Napoli Italy; ^3^ Istituto Diagnostico Varelli Napoli Italy; ^4^ Departments of Obstetrics and Gynecology IRCCS “Burlo Garofolo” Trieste Italy

**Keywords:** 16p12.2 microdeletion, bifid cardiac apex, multiple anomalies, prenatal ultrasound, spongiform cardiomyopathy

## Abstract

**Key Clinical Message:**

From a literature review, this is the first case of fetal 16p12.2 microdeletion syndrome inherited from a normal father with autopsy description and evidence of spongious cardiomyopathy. First trimester intake of doxycycline could be a cofactor.

**Abstract:**

Prenatal diagnosis of a 16p12.2 microdeletion, inherited from normal father, is reported in a dysmorphic 20 weeks fetus. Histopathological examination of the myocardium (not present in the 65 cases in literature) showed bifid apex of the heart and spongiotic structure. Correlation between the deleted genes and cardiomyopathy is discussed.

## Graphical abstract

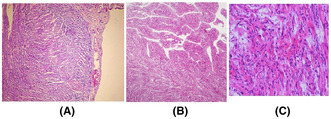

## 1 INTRODUCTION

The 16p12.2 microdeletion is associated with a variable clinical phenotype characterized by intellectual disability, cognitive impairment (sometimes very mild or inapparent), growth retardation, cardiac abnormalities, and other less constant signs. We report a case where 16p12.2 microdeletion in a fetus, inherited from a phenotypically normal father with no intellectual deficit or psychiatric disorders, with marked growth retardation and cardiomyopathy. The 16p12.2 microdeletion can be silent and/or can be present in a completely normal parent, as the father in our case.^1^, ^2^, ^3^ According to Girirajan et al (2018) families with 16p12.2 deletion frequently have a history of neuropsychiatric disorders; in the casuistry of the author, 10 of 42 probands with recurrent microdeletion had another large (> 500 Kb) copy number variation (CNV) elsewhere in the genome. This is not the only type of CNV that can be transmitted by a normal parent; penetrance could depend on a second CNV or other genetic modifiers elsewhere in the genome.

To the best of our knowledge, the present microdeletion is the smallest in the literature associated with an abnormal phenotype, being only 367 Kb compared with the recurrent 520 Kb associated with the syndrome.^4^

Another element of interest is the particular type of cardiomyopathy found on histopathological examination: heart with bifid apex and noncompact appearance of myocardium.^5^, ^6^

## 2 CASE REPORT

The malformed fetus was the first nonabortive pregnancy of clinically healthy and nonconsanguineous Italian parents after four spontaneous abortions in first trimester. The familial anamnesis was negative for genetic disease and malformative syndromes. The father is healthy carrier of beta‐thalassemia. Because of polyabortivity, molecular screening of the main thrombophilic mutations was made in the pregnant and evidenced the presence of compound heterozygosity C677T/A1298C of the MTHFR gene associated with a homozygous PAI‐1 mutation.

The pregnancy history was positive by intake of Bassado© (Doxiciclina) 200 mg during gestational age 2–4 weeks due to a Gardnerella and ureaplasma infection found in the molecular cervicovaginal swab.

**FIGURE 1:**
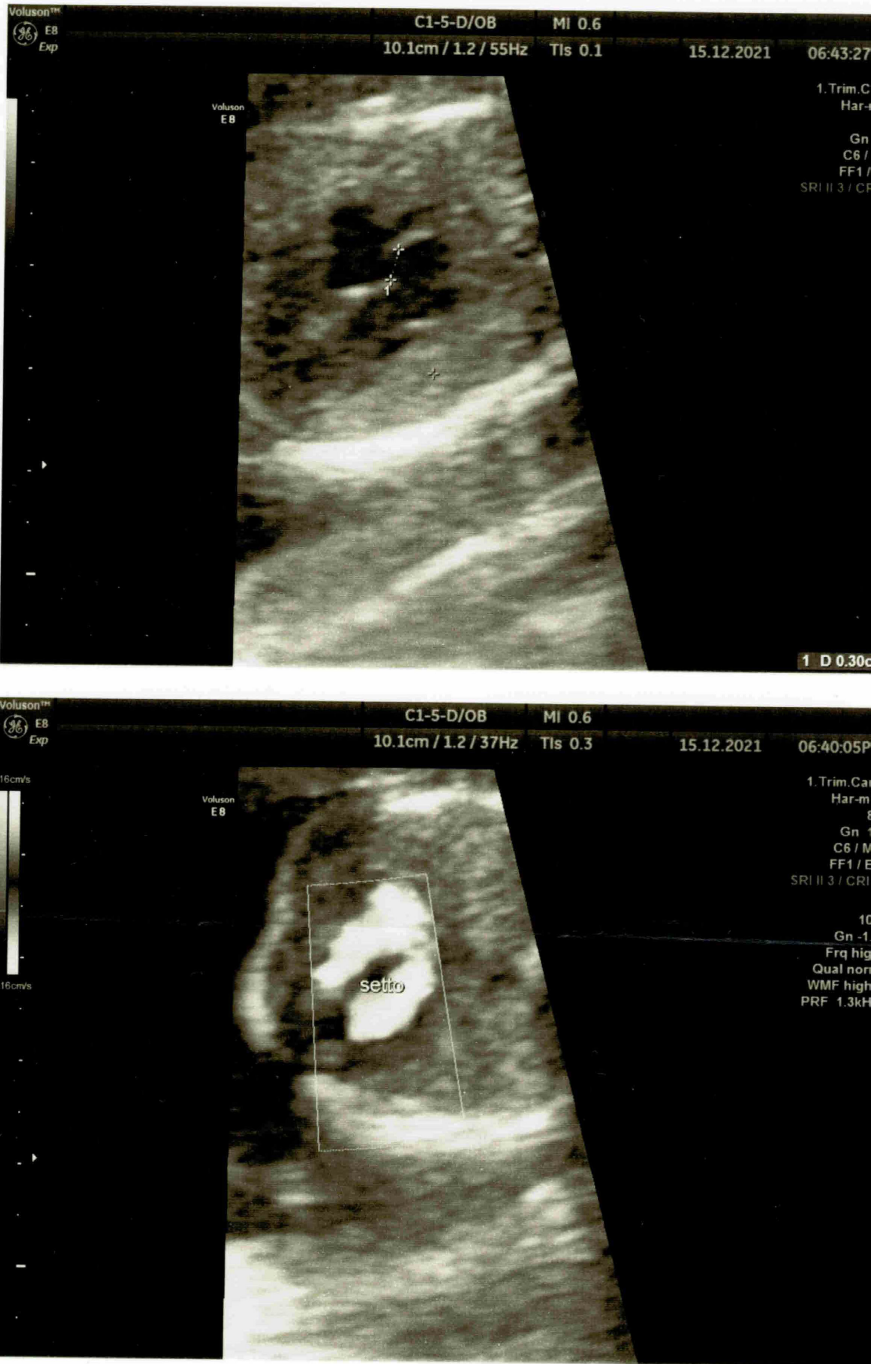
The fetus was small for gestational age, and cardiac anomaly was evidenced consisting in large foramen ovale with suspected ostium primum defect, small penis, altered uterine Doppler velocimetry (RI 0.92) with protodiastolic notch and absent diastolic flow at umbilical artery velocimetry.

In the second trimester, the systemic autoimmunity (anticardiolipin ACA, antigliadin antibodies AGAs) was negative, the homocysteine and glycosylated hemoglobin dosage was in the normal range and the BMI indicated degree II obesity (35.5; range 30–35; W = 83 kg; H = 1.58 m). At prenatal ultrasound in the second trimester of gestation (Figure 1), the fetus was small for gestational age and cardiac anomaly was evidenced consisting in large foramen ovale with suspected ostium primum defect, small penis, altered uterine Doppler velocimetry (RI 0.92) with protodiastolic notch and absent diastolic flow at umbilical artery velocimetry.

Standard and molecular karyotype amniocentesis was performed because of ultrasound abnormalities. The standard cytogenetic Karyotype with a banding resolution of 400 bands revealed a normal male karyotype 46, XY. Chromosomal SNP microarray analysis on the fetal DNA evidenced: arr[GRCh37]16p12.2(21,599,125–219,668,699)x1, that is an heterozygous deletion with an extension of about 367 kb, present at the level of the cytoband p12.2 on chromosome 16, with an interval from nucleotide 21,599,125 to nucleotide 21,966,869. The deletion involved the OTOA [OMIM #607038] and UQCRC2 [OMIM #191329] genes. QPCR analysis in both parents of the 16p12.2 microdeletion revealed the presence of the same microdeletion in the phenotypically normal father.

Considering the ultrasound abnormalities associated with microdeletion at the SNP array and fetal severe growth retardation, the parental couple opted for the termination of pregnancy at the 22nd week of gestation.

## 3 SNP ARRAY ANALYSIS

Array based diagnosis of fetal unbalanced chromosome abnormalities has been successfully employed on prenatal material. The SNP‐arrays are chips containing specific probes for single nucleotide polymorphisms (SNPs) distributed uniformly along the whole genome.

The SNP‐arrays, unlike the classic CGH‐arrays, allow in addition to the detection of CNV, also to determine the genotype corresponding to the single SNP. From the analysis of the distribution of the allele frequency of all the SNPs of the subject under examination it is possible to evaluate the presence of any uniparental disomia (UDP), loss of heterozygosity (LOH, loss of heterozygosity) of certain chromosomal regions and to detect the genetic identity of the descendants (parental consanguinity) useful in the evaluation of autosomal recessive diseases. SNP‐arrays also allow the detection of polyploidies, chimerisms, and mosaicisms by evaluating the presence of an anomalous number of alleles for different SNPs. The Illumina HumanCytoSNP‐12 BeadChip® with BeadArray technology is used, capable to determine the most frequent syndromes, by analyzing ~300,000 SNPs in ~250 disease regions, including subtelomeric, pericentromeric, and sex chromosome regions by studying over 800 genes.

## 4 PATHOLOGICAL FINDINGS ON THE FETUS AND PLACENTA

**FIGURE 2:**
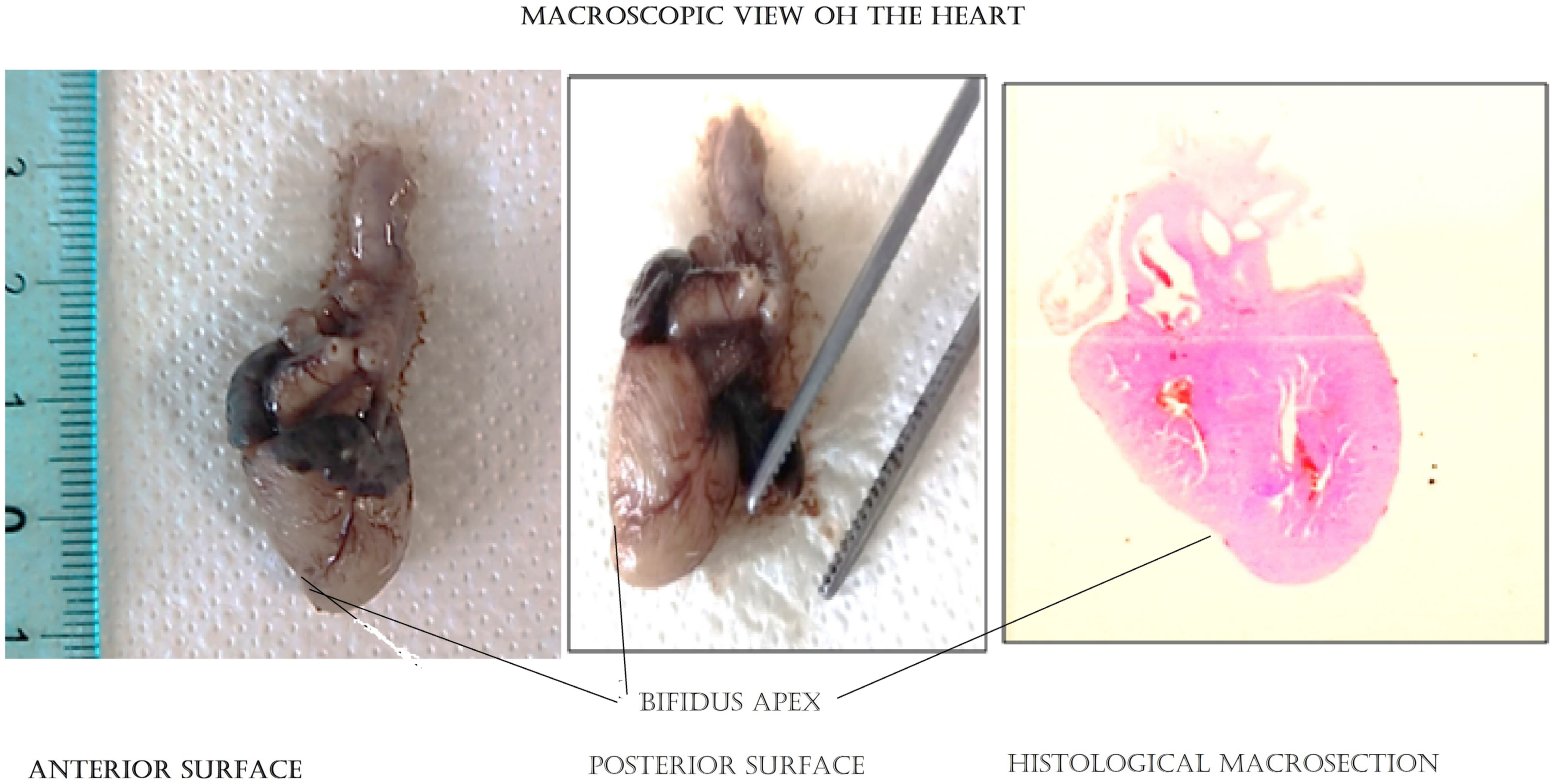
Macroscopic examination of the heart evidenced a bifid apex and normal emergence of cardiac peduncle vessels.

**FIGURE 3:**
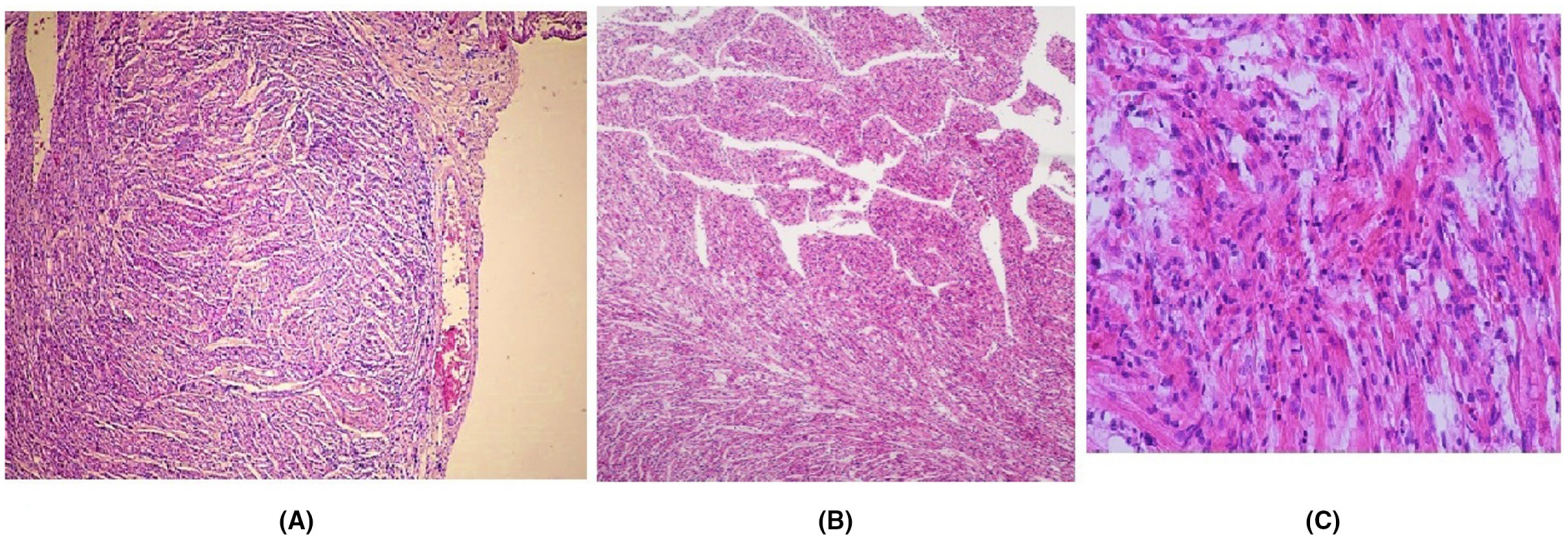
(A) Disordered arrangement of myocardial cells with fissures between myocardial fiber (E–E 20×). (B) Hypetrophic appearance of fissured left ventricular myocardium (E–E 40×). (C) Myocardial fibers with clear changes in the volume and shape of the myocardial fibers, showing marked disarrangement in their histological organization (E–E 200×).

Fetal somatic growth parameters were referable to the 19‐20th w (length 23 cm; weigth 230 g), with a gap of 2–3 weeks compared to the time of amenorrhea. Dysmorphic facies with saddle nose and micrognathia was apparent; male genitalia with small penis was confirmed, and no other gross anomaly was evident on external macroscopic examination. At the thoracoabdominal opening normal topographic anatomy except for thymic hypoplasia was apparent. Histological characteristics of the parenchymatous organs were consistent with the time of fetal development; the observed presence of the hepatic myeloid metaplasia is a normal aspect of fetal liver. Macroscopic examination of the heart (Figure 2) evidenced a bifid apex and normal emergence of cardiac peduncle vessels. At histological examination (Figure 3) the combination of two elements was evident: a spongiotic structure of the myocardium (noncompact myocardium) and architectural disorder with aspects of primary hypertrophic cardiomyopathy (disarrangement).^5^ At skull opening, the conformation of cranial base and fossae were normal. The brain was edematous, smooth without convolutions, with gross features consistent with gestational age.

Placenta weighing 120 g and 9 cm in diameter with an hyperspiralized cord and velamentous insertion. Histopathologic examination excluded multifocal chorangiomatosis (MC), known factor of fetal abnormalities and growth retardation.^7^

## 5 CASUISTRY REVIEW AND DISCUSSION

It is highly probable that the fetal plurimalformative syndrome of our case is correlated to microdeletion. The presence of heart disease, growth retardation, and micropenis is a syndromic association strongly suggestive of a microdeletion syndrome; all three elements are found in the microdeletion 16p12.2 syndrome. Obviously we cannot comment on the presence of mental retardation which is sometimes the only sign, nor can we have information on sensory deficits, in particular hearing impairment. It cannot be excluded that the intake of doxycycline in the organogenesis period constituted an epigenetic factor for the expression of the chromosomal anomaly identically present in the normal father.

Although seven genes of interest (UQCRC2, PDZD9, MOSMO, VWA3A, EEF2K, POLR3E, and CDR2) are within the 520 Kb recurrent deletion in the literature; however, no point mutation or deletion of any of these genes reproduce the phenotype of the syndrome. Since it is a heterozygous microdeletion, the mutation of a gene should be pathogenetic even in heterozygous conditions and not necessarily in the homozygous state. In our case, the deletion is smaller than the larger and more frequently encountered one of 520 Kb and concerns only two genes (OTO A and UQCRC2) deleted in heterozygosity.

The OTOA (OTOAncorin) encodes otoancorin, which belongs to a group of noncollagenous glycoproteins of the acellular gels of the inner ear in mammals. Point mutations can cause sensorineural hearing loss in the homozygous state. The UQCRC2 (ubiQuinol‐cytochrome c reductase core protein II) gene encodes a subunit of mitochondrial complex III; the homozygous mutations of the gene can give neonatal acidosis with hyperammonemia and hypoglycemia. It is to be considered that the mutations could have a milder effect than the deletion involving complete loss of the gene.

Maternal thrombophilia and the intake of doxycycline during pregnancy may have contributed to the pathological phenotype of the fetus. Doxycycline falls into category D (positive evidence of human fetal risk) according to the US FDA pregnancy category.

Generally, patients with 16p12.2 microdeletion have congenital heart disease (CHD), hypoplastic left heart syndrome (HLHS), and bicuspid aortic valve (BAV).^8^ From the literature review, our case with the 16p12.2 microdeletion is the only one documented by autopsy with this particular cardiac phenotype associated with left ventricular noncompaction (LVNC). Left ventricular noncompaction (LVNC) is a very rare congenital cardiomyopathy. It is a disease of endomyocardial trabeculations that increase in number and prominence. This cardiomyopathy carries a high risk of malignant arrhythmias, thromboembolic phenomenon, and left ventricular dysfunction. This disease also has other names like spongy myocardium, spongiform cardiomyopathy, hypertrabeculation, persisting myocardial sinusoids, or zaspopathy.^9^ The mechanisms underlying noncompaction of the ventricular myocardium are still poorly understood; the small GTPase Rac1, with cytogenetic location on 7p22.1 (OMIM #602048) acts as a crucial regulator of numerous developmental events. *Rac1* deficiency in the myocardium impairs cardiomyocyte elongation and organization, and proliferative growth of the heart. A spectrum of CHDs arises in *Rac1* ^*Nkx2.5*^ hearts, implicating *Rac1* signaling in the ventricular myocardium as a crucial regulator of OFT (outflow tracts) alignment, along with compact myocardium growth and development.^5^

Human pathologies related to mutations of the two genes OTOA and UQCRC2 do not lead to cardiac anomalies. However, given the function of the proteins encoded by the two genes, it is possible that, in a particular genetic context, the absence of these proteins could give anomalies in the organization of the myocardium such as that found in our case. Otoancorin is a surface protein^10^, ^11^ that anchors the gel to the surface of the sensory epithelium of the inner ear. Ubiquitinol‐cytochrome c reductase core^10^, ^11^ intervenes in the mitochondrial respiratory chain. The human pathologies reported in the literature are for the mutation of the single gene and not for the contemporary mutation of both genes; therefore, it may be incorrect to compare the combined deletion phenotype of both genes with the mutation phenotype of the single gene.

By consulting the Decipher^12^ database regarding the 16p12.2 microdeletion (21,599,125–21,966,869), analyzing casuistry with an extension microdeletion from 140.20 kb up to 456.55 kb (an interval comparable to our case) a number of 100 cases results. Of these 100, the parental origin is known in 49/100, of which 14/100 paternally inherited, 15/100 maternally inherited, and 1/100 biparental and for the remaining 19/100 the sex of the parent is not known. For 50/100 cases the parental origin is unknown, and 1/100 is de novo with neonatal hypotonia. Among those of parental origin, in 7/100 phenotypic normality is reported both in the parent and in the child; in 4/100 the presence of the severe pathological phenotype in the parent and child is reported. Therefore in the majority of cases (45/49) of parental origin, the carrier parent is normal. Among the most frequent phenotypic anomalies are mental retardation, neurological disorders, sensorineural deafness, and only in one case heart disease. There is no statistically significant difference between the pathological cases with a normal parent with regard to the sex of the parent; therefore, it is possible to exclude an imprinting phenomenon; the imprinting in 16p12.2 region is not known in the literature.^13^

On the basis of the review of the above casuistry and our personal observation, the prenatal diagnosis of microdeletion 16p12.2 with an extension in the range of about 140–450 kb of parental origin with normal ultrasound, by itself is not a sufficient indication for pregnancy termination.

Family communication about genetic risk should consider that the child phenotype may be completely normal or have mild‐to‐medium intellectual disability, attention deficit hyperactivity disorder, and/or sensorineural hearing impairment as the only syndromic signs.

## AUTHOR CONTRIBUTIONS

**Mariano Stabile:** Conceptualization; formal analysis. **Anna F Rispoli:** Investigation; validation; writing – original draft. **Maurizio Capuozzo:** Investigation; validation; writing – review and editing. **Umberto Ferbo:** Investigation; methodology; validation. **Guglielmo Stabile:** Supervision; validation; visualization.

## FUNDING INFORMATION

This research received no external funding.

## CONFLICT OF INTEREST STATEMENT

The authors declare no conflict of interest.

## CONSENT

Written informed consent was obtained from the patient to publish this report in accordance with the journal's patient consent policy.

## Data Availability

The authors confirm that the data supporting the findings of this study are available within the article.
